# Genome-wide transcriptome profiling of human trabecular meshwork cells treated with TGF-β2

**DOI:** 10.1038/s41598-022-13573-8

**Published:** 2022-06-10

**Authors:** Breedge Callaghan, Karen Lester, Brian Lane, Xiaochen Fan, Katarzyna Goljanek-Whysall, David A. Simpson, Carl Sheridan, Colin E. Willoughby

**Affiliations:** 1 Genomic Medicine Group, Biomedical Sciences Research Institute, Ulster University, Coleraine, BT52 1SA Northern Ireland UK; 2 Institute of Life Course and Medical Sciences, University of Liverpool, Liverpool, L7 8TX UK; 3 Translational Radiobiology Group, Division of Cancer Sciences, University of Manchester, Manchester Academic Health Science Centre, Christie NHS Foundation Trust Hospital, Manchester, M20 4BX UK; 4 School of Medicine, Physiology, National University of Ireland Galway, Galway, H91 W5P7 Ireland; 5 The Wellcome – Wolfson Institute for Experimental Medicine, School of Medicine, Dentistry and Biomedical Sciences, Queen’s University, Belfast, UK

**Keywords:** Molecular biology, Diseases, Molecular medicine, Pathogenesis

## Abstract

Glaucoma is a complex neurodegenerative disease resulting in progressive optic neuropathy and is a leading cause of irreversible blindness worldwide. Primary open angle glaucoma (POAG) is the predominant form affecting 65.5 million people globally. Despite the prevalence of POAG and the identification of over 120 glaucoma related genetic loci, the underlaying molecular mechanisms are still poorly understood. The transforming growth factor beta (TGF-β) signalling pathway is implicated in the molecular pathology of POAG. To gain a better understanding of the role TGF-β2 plays in the glaucomatous changes to the molecular pathology in the trabecular meshwork, we employed RNA-Seq to delineate the TGF-β2 induced changes in the transcriptome of normal primary human trabecular meshwork cells (HTM). We identified a significant number of differentially expressed genes and associated pathways that contribute to the pathogenesis of POAG. The differentially expressed genes were predominantly enriched in ECM regulation, TGF-β signalling, proliferation/apoptosis, inflammation/wound healing, MAPK signalling, oxidative stress and RHO signalling. Canonical pathway analysis confirmed the enrichment of RhoA signalling, inflammatory-related processes, ECM and cytoskeletal organisation in HTM cells in response to TGF-β2. We also identified novel genes and pathways that were affected after TGF-β2 treatment in the HTM, suggesting additional pathways are activated, including Nrf2, PI3K-Akt, MAPK and HIPPO signalling pathways. The identification and characterisation of TGF-β2 dependent differentially expressed genes and pathways in HTM cells is essential to understand the patho-physiology of glaucoma and to develop new therapeutic agents.

## Introduction

Glaucoma is a neurodegenerative disease resulting in progressive optic neuropathy and is a leading cause of irreversible blindness worldwide^1^. Primary open angle glaucoma (POAG) is the predominant form accounting for approximately two-thirds of glaucoma patients affecting 65.5 million people globally^2^. The pathogenesis of POAG is multi-factorial^3^ and complex. Elevation of intra-ocular pressure (IOP) has been identified as a significant risk factor for POAG^4^. Multiple international randomised controlled trials have demonstrated that medically or surgically lowering IOP reduces glaucoma progression^5^. Changes to intra-ocular pressure are generated by resistance to fluid outflow in the trabecular meshwork (TM). Outflow resistance is higher in POAG patients^6^ because of cellular and molecular changes in the TM resulting in reduced outflow facility. Despite the prevalence of POAG and the identification of over 120 glaucoma related genomic loci^7^, the molecular mechanisms of POAG are still poorly understood. However, bioinformatic analyses of glaucoma related genes have repeatedly identified the transforming growth factor beta (TGF-β) signalling pathway in the molecular pathology of POAG^8^.

Physiologically, TGF-β2 is produced by the ciliary epithelium and lens epithelium^10^. Multiple studies have identified elevated concentrations of TGF-β2 in the aqueous humour of POAG patients^9,11,12^ however the mechanism of this elevation is unknown^12^. The levels of TGF-β2 in the aqueous humour range from approximately 0.5 ng/ml to 8 ng/ml^11,13^ with the highest levels found in POAG patients^11^. The expression of TGF-β2 is also elevated in the glaucomatous TM and in cultured glaucoma TM (GTM) cells^14^. Adenoviral driven expression of active TGF-β2 elevated IOP and reduced outflow facility in rodent eyes in vivo^15^. Perfusion of TGF-β2 in the ex vivo anterior segment organ culture model resulted in an increase in IOP and reduced outflow facility with an accumulation of extracellular matrix (ECM) material^16^. These ECM changes in the TM mirror those seen in POAG patients^6,17^ and correlated with the degree of axonal damage in the optic nerve^18^. Moreover, there is a significant body of evidence that TGF-β2 drives patho-physiological processes resulting in POAG^8,9,12,19^.

TGF-β2 is a multifunctional cytokine which controls a wide array of cellular processes including cell growth, differentiation, apoptosis, migration, ECM production, immunity and development^20,21^. The function of TGF-β2 is both cell and context specific^20^, and understanding the role of TGF-β2 in driving structural and functional alterations in the outflow pathway is essential to develop new glaucoma therapies based on the modulation of TGF-β2 signalling in the trabecular meshwork^9,19,22^. Three microarray studies have analysed alterations in gene expression induced by TGF-β2 in cultured human HTM cells^16,23,24^; none of these studies made the complete datasets publicly available. All studies identified alterations in ECM and cytoskeletal components, but common genes were limited to v-maf musculoaponeurotic fibrosarcoma oncogene homolog (MAF), plasminogen activator inhibitor-1 (PAI-1) and latent-transforming growth factor beta-binding protein 1 (LTBP1)^16,23,24^. Several key genes previously identified using RT-qPCR for prioritised targets were not detected in these microarray studies, although there is significant evidence that they are regulated by TGF-β2 in the human TM^12,25,26^. At their core, microarrays are simple devices to simultaneously measure the relative concentration of many different RNA sequences and they are not without their limitations^27^. However, RNA-Seq technology offers significant benefits over previous microarray technologies with improved dynamic range, depending on sequence depth, detection of transcript isoforms (splicing) and novel transcripts.^27,28^.

Therefore, the aim of this study was to employ RNA-seq to investigate genome-wide alterations in the transcriptome of normal human donor TM cells stimulated with TGF-β2 and investigate possible pathophysiological mechanisms driving POAG.

## Materials and methods

### Sample collection, preparation, and tissue culture

Cadaveric eyes (n = 5) were provided by the Liverpool Research Eye Bank and approved by the local ethics review board (RETH000833) handled in accordance with the tenets of the Declaration of Helsinki. Eyes were obtained from the Royal Liverpool University Hospital Mortuary and medical history was unknown. Donor eyes were excluded if the maximum post-mortem time exceeded 48 h or there was a history of glaucoma or ocular surgery (Supplemental Fig. 1). TM cells were isolated using the blunt dissection method as reported previously^29^. Cells were maintained in Dulbecco`s Modified Eagle Media (DMEM)-low glucose (Sigma, UK) supplemented with 10% fetal calf serum (Bio Sera, UK), 2 mM l-glutamine (Sigma, UK), Pen/Step (Sigma, UK), and 2.5 µg/mL Fungizone (amphotericin B, Sigma, UK). Samples were incubated at 37 °C (5% CO2 and 95% humidity). TM characterisation was carried out as previously described^29^ and demonstrated upregulated myocilin protein expression in response to dexamethasone treatment (Supplemental Fig. 1B). Briefly, proteins from the dexamethasone treated and untreated donors were isolated using RIPA buffer(Thermo) and were separated on a 4–20% SDS-PAGE gel (Biorad; Uk). Proteins were transferred to a nitrocellulose membrane (Bio-rad; Uk) using the trans-blot cell system (Bio-rad). Membranes were blocked in 5% nonfat dry milk and incubated with a polyclonal rabbit anti-myocilin primary antibody (kind gift from Dr. W. Daniel Stamer) or GAPDH (CST, UK) overnight at 4 °C. Membranes were washed and incubated with horseradish peroxidase-conjugated secondary antibodies in TBS-T containing 5% milk. Protein-antibody complexes were detected using chemiluminescence (SuperSignal West Pico PLUS Chemiluminescent Substrate, Thermo Scientific) in a ChemiDoc XRS + imaging system (Bio-Rad).

### TGF-β2 stimulations

Human TM cells between passages 5 and 7 were grown to 80% confluence and growth arrested using serum free medium prior to stimulation. Cells were stimulated with recombinant human TGF-β2 (R&D Systems, UK) at a concentration of 5 ng/mL for 24 h. Vehicle control cells were stimulated with equal volumes of 4 mM HCl and 0.1% BSA solution (Sigma, UK). The viability of TM cells treated with TGF-β2 was assessed using the 3-(4,5-dimethylthiazol-2-yl)-2,5-diphenyltetrazolium bromide assay (MTT, Sigma-Aldrich, USA) (Supplemental Fig. 1C). 24 h post TGF-β2 treatment, 10uM of MTT solution was added to each well and incubated for 3 h. After incubation, the media was removed and the formazan crystals were dissolved in 100 µl of DMSO. The optical densities (OD) of the dissolved formazan crystals was read on a plate reader at 570 nm (Omega FluroStar; US). The quantification of cell viability was obtained by comparing the optical density of the treated and untreated samples. The relative cell viability was calculated for each tissue as Arbitral Unit (AU), extrapolated by Optical Density (OD) of the samples.

### RNA-seq of TGF-β2 stimulated TM cells

Total RNA from human culturedTM cells was isolated using the Qiagen Universal All Prep (Qiagen, UK) kit as per manufacturer’s specifications. Total RNA was quantified on the Nanodrop-1000 (Thermofisher, UK), and quality was determined by the Bioanalyser 2100 (Agilent, UK). All RNA sequencing experiments were conducted at Exiqon Services, Denmark. Two groups of mRNA libraries were prepared: a group of 5 control human TM samples (Donor Control) and a group of 5 treated TM samples (Donor Treated). After extracting the total RNA, mRNA was enriched using the oligoT bead system and the isolated mRNA was enzymatically fragmented. First and second strand synthesis were performed, and the double stranded cDNA was purified (AMPure XP, Beckman Coulter, Denmark). The cDNA was end repaired, 3’ adenylated and Illumina sequencing adaptors ligated onto the fragments ends. Following this the mRNA stranded libraries were pre-amplified with PCR and purified (AMPure XP). Library size distribution was validated, and quality was inspected on a Bioanalyser high sensitivity DNA chip (Agilent Technologies, UK). High quality libraries were quantified using q-PCR, the concentration normalised, and the samples pooled according to the project specification (number of reads). The library pools were re-quantified with q-PCR and optimal concentration of the library pool was used to generate the clusters on the surface of a flowcell before sequencing on a Nextseq 500 instrument using a High Output sequencing kit (51 cycles) according to the manufacturer instructions (Illumina Inc., USA).

### RNA-Seq data analysis

Following sequencing, intensity correction and base calling (into BCL files), FASTQ files were generated using appropriate bcl2fastq software (Illumina Inc.) which includes quality scoring of each individual base in a read. Data was separated for paired end reads to determine whether the second read significantly differs from the first in terms of overall quality. Data analysis was performed by Exiqon (Exiqon, Denmark). The components of Exiqon NGS RNA-Seq analysis pipeline include Bowtie2 (v.2.2.2), Tophat (v2.0.11) and Cufflinks (v2.2.1). As we were comparing groups, Cuffdiff, normally used for unpaired samples to calculate FPKM (number of fragments per kilobase per million mapped fragments) was replaced with featureCounts. FeatureCounts was used to calculate the counts of mapped reads in specific genes when groups with paired samples were compared, and generalised linear model likelihood ratio test (glmLRT) implemented by edgeR was used to test differential expression across submitted samples using featureCounts input. Post processing of Cufflinks and Cuffdiff was performed using CummeRbund and Bioconductor software to generate visual representations of sequencing results. The raw RNA-Seq data was deposited and released in the SRA database (Study: PRJNA820984; Accessions:SAMN27032229).

### Bioinformatic analysis of biological process and pathways

Following analysis by Exiqon (Denmark), further functional analysis was performed through Database for Annotation, Visualization and Integrated Discovery (DAVID) bioinformatic package, (available at http://david.abcc.ncifcrf.gov) and Ingenuity Pathway Analysis (IPA)^30,31^. Gene Ontology (GO) enrichment analysis was carried out to investigate relationships between the significantly expressed genes and their cellular compartment, biological processes and molecular function. Significance was calculated in DAVID using the Fisher’s exact test with the Benjamini and Hochberg algorithm applied to obtain corrected p-values. Only terms with a Benjamini-corrected p-value < 0.01 were considered significant. IPA v01-08 (Qiagen, UK) Core and Comparison analyses were performed with lists of differentially expressed genes (DEGs) generated from expression analyses that included gene name, false discovery rate (FDR) p-value and log fold change (FC). A FDR p-value < 0.025 was used to select lists of DEGs for analysis. Core analysis output in IPA included ranked lists of canonical pathways and single molecule upstream regulators of a single DEG supplied. The canonical pathways and upstream regulators identified in two or more core analyses in IPA were compared in comparison analyses. Comparisons were performed by hierarchical clustering based on process enrichment score or activation z-score. Fisher’s exact tests reported as a likelihood value (− log(p-value)) were performed to assess canonical pathway enrichment. Canonical pathways and upstream regulators were ranked by activation z-score which is a weighted statistic that correlates measured gene expression with the expected direction of expression of the DEG list supplied. The activation score is equivalent to a zero centred normal distribution z-score therefore only scores of ± 1.96 were regarded as significant and the sign of the z-score indicates the direction of regulation of the process.

The top list of DEG were added to the STRING database to obtain the protein–protein interactions (PPI) (medium confidence 0.400) viewed on the Cytoscape software (Cytoscape 2.8.3 (http://www.cytoscape.org)). Candidate genes were calculated using MCODE to create clusters followed by the ClueGo/CluePedia and KEGGscape plugins, which visualises the important biological processes and pathways for clusters of genes in a functional grouped network^32^.

### RNA-Seq data validation

Validation of RNA samples was performed on the same TM donor cells used in the RNA-seq. Using Primer Design Ltd primer assays (Primer Design Ltd, UK). RT-qPCR was performed for selected significantly altered DEGs. 1ug of total RNA was reverse transcribed into cDNA using miScript II RT (Qiagen, UK) kit according to manufacturers’ specifications (Qiagen, UK). Real time analysis was performed using custom primers (Primer Design Ltd. UK) for target genes (Supplemental Table S1). RT-qPCR was performed on a LightCycler®480 real-time PCR system (Roche Diagnostics, Switzerland). All mRNA was measured at CT threshold levels and normalised with the average CT values of a reference gene; GAPDH. Values were expressed as fold increase over the corresponding values for control by the 2-ΔΔCT method.

### Statistical analysis

Two independent experiments were performed, and the average (± SD) results were calculated using GraphPad software (GraphPad Software, San Diego, USA). Data were expressed as the mean values ± SD and graphed using log scale. Statistical significance was analysed using a student t-test. Differences in the mean were considered statistically significant if p < 0.05.

## Results

### Descriptive features of RNA-Seq data

Two mRNA libraries (donor control and donor treated) were sequenced on a flow cell using NexSeq500 (Illumina. Inc). A total of 30 million 50 bp paired end reads were obtained and on average 46.3 million reads were obtained from each sample ranging from 40 to 57 million reads per sample (Supplemental Table S1). Paired-end reads were separated to determine whether the second read significantly differed from the first in overall quality. Majority of the data had a Q score greater than 30 (> 99.9% correct). Mapping of sequencing data represents a useful QC step in RNA-Seq analysis pipeline as it can help evaluate the quality of samples. The genome mapping for each sample was on average 82% and the uniformity of the mapping results suggests that the samples are comparable (Supplemental Table S2). A principal component analysis (PCA) was carried out to identify the main components of variation in the data set using the R package, prcomp (data not shown).

### Differential gene expression

**Figure 1:**
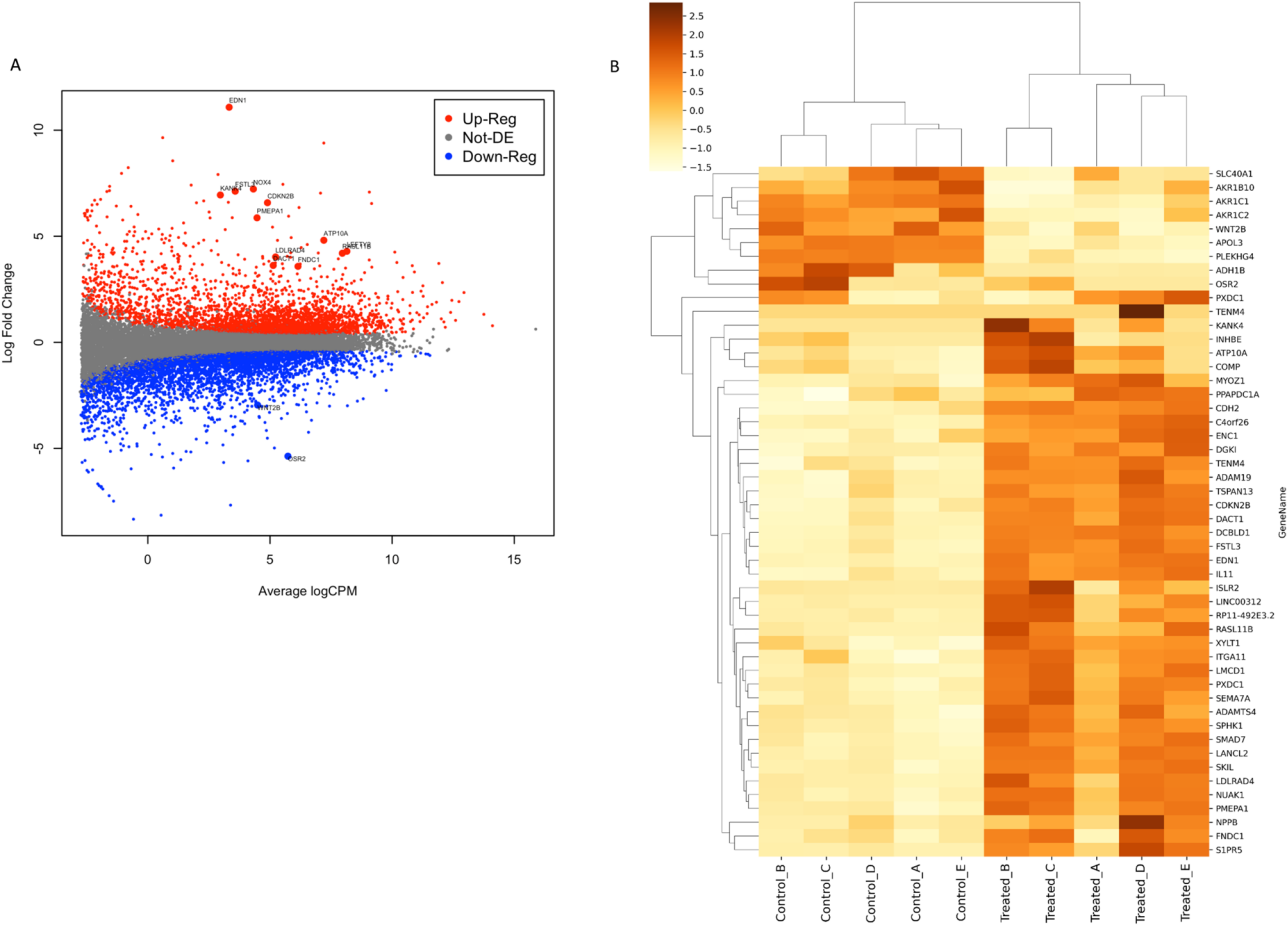
Gene expression analysis of TM cells treated with TGF-β2. (**A**) MA Plot showing the relationship between the LogFC and the logCPM. Red dots = significantly (p < 0.05) up-regulated genes between the two sample with a fold change(FC) > 2; Grey dots = genes were not differentially (p > 0.05) expressed between the two sample groups with a FC < 2; Blue dots = significantly (p < 0.05) down-regulated genes between the two samples with a FC > − 2. (**B**) Hierarchical clustering by sample and transcripts was performed on all samples passing the QC using the top 50 DEGs that have the largest coefficient of variation based on FPKM counts.

To determine changes in gene expression, primary TM cells were treated with 5 ng TGF-β2 and analysed using RNA-Seq. A total of 17,186 genes were differentially expressed compared to vehicle controls. The expression of 10,106 genes were found to be statistically significantly (p < 0.05) altered. All data points were graphed using a MAplot (Fig. 1A) which allowed enhanced and distinguishable expression visualisation. A selection criterion based on a statistical significance of p < 0.05 and logFC greater than 2 was applied, as visualised by the red (upregulated) and blue (downregulated) dots on the MAplot. To discriminate distinguishable expression patterns specific to the donors included in this study, the top 50 DEGs were graphed using a two-way hierarchical cluster using a Pearson Correlation distance algorithm and a complete agglomeration method with gene wise scaled and centred log transformed FPKM count data (Fig. 1B). Each donor responded to the concentration of TGF-β2 used in the study. The heat map (Fig. 1B) shows the individual donor responses to TGF-β2 and shows variability. All donors were male and aged between 57 and 65 years of age. The basis of the donor biological variability and TGF-β2 responsiveness for specific DEGs is not known and may relate to individual donor characteristics or epigenetic factors. The top 50 most up- and down-regulated genes were ranked by log FC and are shown in Tables 1 and 2, respectively.

**Table 1:** Top 50 up-regulated genes.

Gene ID	Gene name	LogFC	p-vlaue	FDR
KANK4	KN Motif and Ankyrin Repeat	11.08	3E−95	9E−92
LINC00312	Long Intergenic Non-Protein Coding RNA 312	9.65	1E−44	5E−42
C4orf26	Chromosome 4 Open Reading Frame 26	9.39	7E−45	3E−42
ISLR2	Immunoglobulin Superfamily Containing Leucine	7.91	3E−80	6E−77
LDLRAD4	Low Density Lipoprotein Receptor Class A Domain Containing 4	7.23	2E−59	2E−56
RASL11B	RAS Like Family 11 Member B	7.12	3E−60	3E−57
IL11	Interleukin 11	7.08	5E−45	2E−42
LEFTY2	Left–Right Determination Factor 2	6.94	5E−62	7E−59
EDN1	Endothelin 1	6.58	6E−150	1E−145
LMCD1	LIM And Cysteine Rich Domains 1	6.35	2E−49	1E−46
S1PR5	Sphingosine-1-Phosphate Receptor 5	6.14	3E−56	3E−53
NOX4	NADPH Oxidase 4	5.87	2E−105	2E−101
NPPB	Natriuretic Peptide B	5.86	6E−50	4E−47
PMEPA1	Prostate Transmembrane Protein Androgen Induced 1	4.81	3E−84	7E−81
SEMA7A	Semaphorin 7A	4.61	9E−84	2E−80
CDKN2B	Cyclin Depended Kinase Inhibitor 2B	4.29	1E−92	4E−89
FSTL3	Folistatin-Like 3	4.20	3E−99	1E−95
MYOZ1	Myozenin 1	4.13	5E−55	3E−52
INHBE	Inhibin Beta E Subunit	4.13	2E−43	6E−41
DGKI	Diacylglycerol Kinase Iota	4.05	3E−45	1E−42
FNDC1	Fibronectin Type III Domain-Containing Protein 1	4.02	7E−57	6E−54
TLL2	Tolloid-like 2	3.92	3E−53	2E−50
XYLT1	Xylosyltransferase 1	3.88	4E−55	3E−52
COMP	Cartilage Oligomeric Matrix Protein	3.82	8E−47	3E−44
TSPAN13	Tetraspanin 13	3.75	5E−43	2E−40
NUAK1	NUAK Kinase Family Member 1	3.69	3E−48	1E−45
ATP10A	ATPase Phospholipid Transporting 10A	3.62	4E−66	6E−63
DACT1	Dishevelled Binding Agonist of Beta Catenin 1	3.58	3E−58	3E−55
CDH2	Cadherin 2	3.50	5E−44	2E−41
TENM4	Teneurin Transmembrane Protein 4	3.47	9E−58	9E−55
ADAMTS4	ADAM Metallopeptidase with Thrombospondin Type 1 Motif 4	3.27	7E−55	5E−52
SPHK1	Sphingosine Kinase 1	3.10	9E−62	1E−58
LANCL2	LanC-like 2	3.01	4E−55	3E−52
PPAPDC1A	Phosphatidic Acid Phosphatase Type 2 Domain-Containing Protein 1A	2.99	4E−55	3E−52
ITGA11	Integrin Subunit Alpha 11	2.96	4E−45	1E−42
ADAM19	ADAM Metallopeptidase Domain 19	2.89	8E−49	4E−46
PXDC1	PX Domain Containing 1	2.62	9E−49	5E−46
ENC1	Ectodermanl-Neural Cortex 1	2.50	6E−47	3E−44
DCBLD1	Discoidin, CUB, And LCCL Domain Containing 1	2.46	6E−48	3E−45
SMAD7	SMAD Family Member 7	2.45	1E−44	4E−42
SKIL	SKI-like Proto-Oncogene	2.19	1E−47	7E−45

**Table 2:** Top 50 down-regulated genes.

Gene ID	Gene name	LogFC	p-vlaue	FDR
ADH1B	Alcohol Dehydrogenase 1B	− 7.66	3E−102	2E−98
STEAP4	STEAP Family Member 4	− 5.53	1E−30	2E−28
OSR2	Odd-Skipped Related Transcription Factor 2	− 5.37	4E−67	7E−64
APOL3	Apolipoprotein L3	− 4.35	5E−46	2E−43
AKR1B10	Aldo–Keto Reductase Family 1 Member B10	− 4.24	2E−51	1E−48
SLC2A12	Solute Carrier Family 2A Member 12	− 4.16	1E−31	2E−29
IFIT2	Interferon Induced Protein With Tetratricopeptide Repeats 2	− 3.84	3E−38	8E−36
AKR1C1	Aldo–Keto Reductase Family 1 Member C1	− 3.67	3E−49	2E−46
TMEM140	Transmembrane Protein 40	− 3.65	8E−34	1E−31
PSMB9	Proteosome Subunit Beta 9	− 3.64	2E−37	4E−35
COL21A1	Collagen Type XXI Alpha 1 Chain	− 3.62	5E−39	1E−36
SLC40A1	Solute Carrier Family 40 Member 1	− 3.59	3E−48	2E−45
IFIT3	Interferon Induced Protein With Tetratricopeptide Repeats 3	− 3.54	1E−40	3E−38
AKR1C3	Aldo–Keto Reductase Family 1 Member C3	− 3.35	2E−36	4E−34
LINC00341	Spectrin Repeat Containing Nuclear Envelope Family Member 3	− 3.31	1E−35	3E−33
AKR1C2	Also-Keto Reductase Family 1 Member C2	− 3.30	4E−65	6E−62
PLEKHG4	Pleckstrin Homology and RhoGEF Domain Containing G4	− 3.01	5E−47	2E−44
PLEKHA6	Pleckstrin Homology Domain Containing A6	− 2.96	1E−30	2E−28
WNT2B	Wnt Family Member 2B	− 2.96	3E−56	3E−53
PTX3	Pentraxin 3	− 2.93	5E−29	7E−27
DDX60	DExD/H-Box Helicase 60	− 2.80	5E−43	2E−40
TOP2A	DNA Tooisomerase II Alpha	− 2.74	2E−35	4E−33
SEMA3D	Semaphorin 3D	− 2.71	1E−28	1E−26
CSF1	Colony Stimulating Factor 1	− 2.70	5E−30	7E−28
RAB27B	RAB27B, Member RAS Oncogene Family	− 2.69	7E−32	1E−29
PARP10	Poly(ADP-ribose) polymerase Family Member 10	− 2.62	2E−35	5E−33
DUSP6	Dual Specificity Phosphatase 6	− 2.49	3E−28	3E−26
GMPR	Guanosine Monophosphate Reductase	− 2.47	8E−29	1E−26
SMAD3	Smad Family Member 3	− 2.46	2E−33	3E−31
PHLDA1	Pleckstrin Homology Like Domain Family A Member 1	− 2.42	2E−39	5E−37
ISYNA1	Inositol-3-Phosphate Synthase 1	− 2.31	6E−35	1E−32
S1PR3	Sphinosine-1-Phosphate Receptor 3	− 2.25	1E−42	4E−40
ADM	Adrenomedullin	− 2.24	3E−37	6E−35
PARP14	Poly(ADP-ribose) polymerase Family Member 14	− 2.23	2E−28	3E−26
UBE2L6	Ubiquitin Conjugating Enzyme E2 L6	− 2.17	3E−41	8E−39
ZFP36	Zinc Finger Protein 36	− 2.13	1E−31	2E−29
NABP1	Nucleic Acid Binding Protein 1	− 1.98	6E−38	1E−35
TENC1	Tensin Like C1 Domain Containing Phosphatase	− 1.98	4E−30	5E−28
ALDH3B1	Aldehyde Dehydrogenase 3 Family Member B1	− 1.92	9E−34	2E−31
CPA4	Carboxypeptidase A4	− 1.83	3E−29	5E−27
MOV10	MOV10 RISC Complex RNA Helicase	− 1.68	2E−29	3E−27

### Functional enrichment analysis of the differentially expressed genes

**Figure 2:**
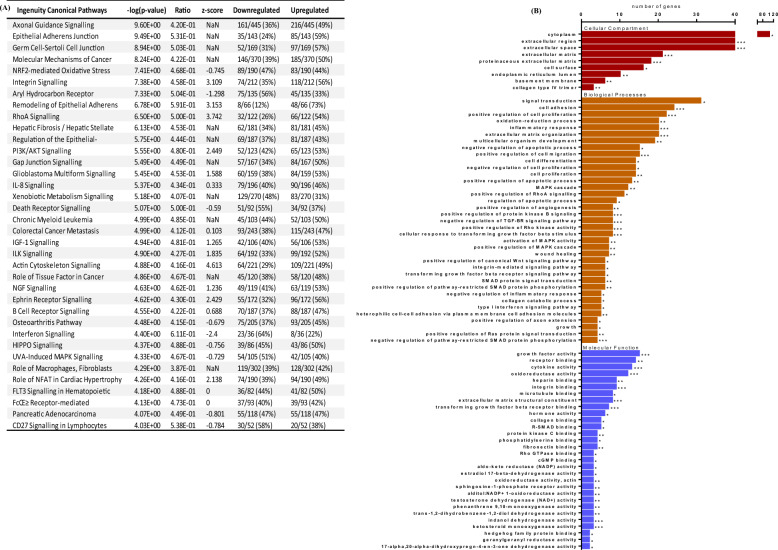
Gene Ontology and canonical pathway analysis. (**A**) A table showing significant biological processes and canonical pathways from IPA for the differentially expressed genes found between donor control and TGFβ2 donor treated. (**B**) Gene Ontology (GO) analysis of the top 50 upregulated and downregulated genes investigating the cellular compartment, biological processes, and molecular functions of the top 50 upregulated and 50 downregulated genes analysed using the DAVID functional process on R. *p < 0.01, **p < 0.001, ***p < 0.0001.

Canonical pathways are biological cascades that are well-defined and are responsible for the inducing a specific consequential function in a cell in response to a stimulus or biological process. In this study, we performed a canonical pathway analysis (Fig. 2A), with the use of the Ingenuity Pathway Analysis (IPA, Qiagen, UK)^31^ using the DEG, with a FDR p-value < 0.025. A total of 254 IPA canonical pathways were significantly enriched as determined using a Fisher’s Exact Benjamini Hochberg test. The top ranked canonical pathways with an enrichment score > 4 (Fisher’s Exact BH adjusted p < 0.0001) were chosen for enrichment analysis.

To ascertain the potential role and function of the top DEG after TGF-β2 treatment, a GO annotation and enrichment analysis was carried out using DAVID software, on the DEG listed in Tables 1 and 2, assessing significant biological processes, cellular compartment, and molecular function (Fig. 2B). This type of analysis differs from canonical pathway analysis as it characterises functional relationships between genes that have been documented in the previous literature. Overall, a total of 75 significant processes were found to be regulated by different combinations of the top 50 most up- and down-regulated genes. The DEGs were predominantly enriched and categorised into ECM regulation (20%), TGF-β signalling (7%), SMAD signalling (7%), proliferation/apoptosis (5%), inflammation/wound healing (5%), MAPK signalling (5%), oxidative stress (3%) and RHO signalling (2%). Interestingly, common terms associated with the DEGs, between the set of DAVID and IPA analysis, include RhoA signalling, ECM, cytoskeletal organisation and inflammatory-related processes, highlighting their potential significance in TM cells in response to TGF-β2.

**Figure 3:**
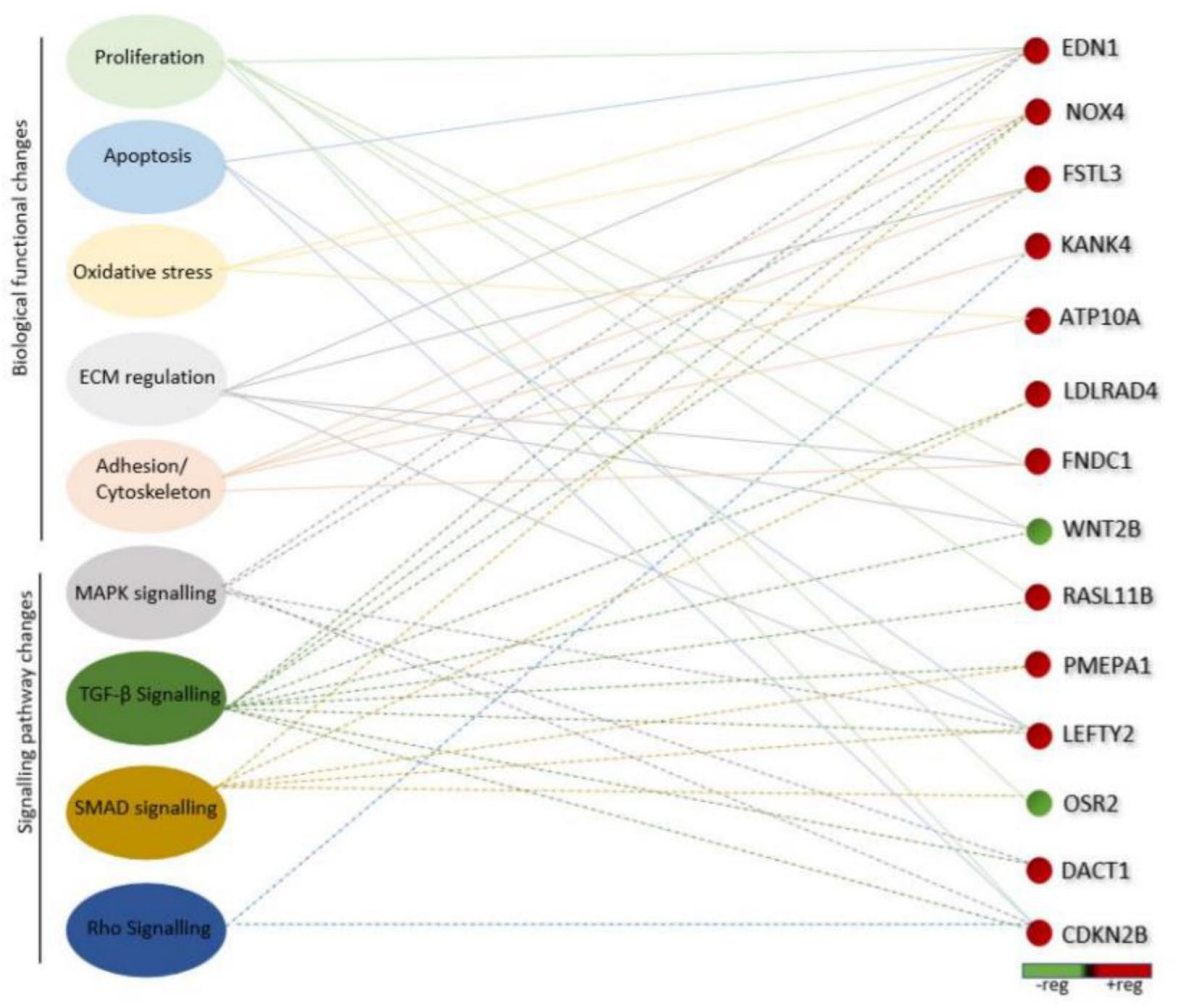
A total of 14 genes were linked together via biological pathways and/or functions that are directly related to glaucoma or TGFβ signalling. The STRING database on the Cytoscape software was used to visualise interactions between upregulated (red) and downregulated (green) genes, with a fold change > 2 and p value < 0.05. Candidate genes were calculated using MCODE to create clusters followed by the ClueGo/CluePedia and KEGGscape plugins, which visualises the important biological processes and pathways for clusters of genes in a functional grouped network. The results were graphed to depict the following: solid lines indicate a relationship between the identified gene and nominated biological functions while dotted lines connect genes to signalling pathways known to play a role in POAG.

To determine potential candidate genes involved in the canonical and enriched pathways associated with the pathogenesis of POAG and TGF-β2 signalling, an analysis of the genes reoccurring within the GO and canonical pathway analyses was carried out using ClueGo/CluePedia plug-in for the Cytoscape software. A total of five biological functional changes including proliferation, apoptosis, oxidative stress, ECM regulation and adhesion/cytoskeleton in combination with four signalling pathway changes including MAPK signalling, TGF-β signalling, SMAD signalling and RHO signalling were used as the selection criteria (Fig. 3). From this analysis, we identified a role for the upregulated genes endothelin 1 (EDN1), follistatin-like protein 3 (FSTL3), KN motif and ankyrin repeat domain-containing protein4 (KANK4), ATPase phospholipid transporting 10A (ATP10A), low density lipoprotein receptor class A domain-containing 4 (LDLRAD4), fibronectin type 3 domain-containing 1 (FNDC1), RAS-like family 11 member B (RASL11B), prostate transmembrane protein, androgen induce 1 (PMEPA1), left right determination factor 2 (LEFTY2), dishevelled binding antagonist of beta catenin 1 (DACT1), and cyclin dependent kinase inhibitor 2B (CDKN2B). Wnt family member 2B (WNT2B), and odd-skipped related transcription factor 2 (OSR2) were downregulated genes.

### Validation of differentially expressed genes by RT-qPCR

**Figure 4:**
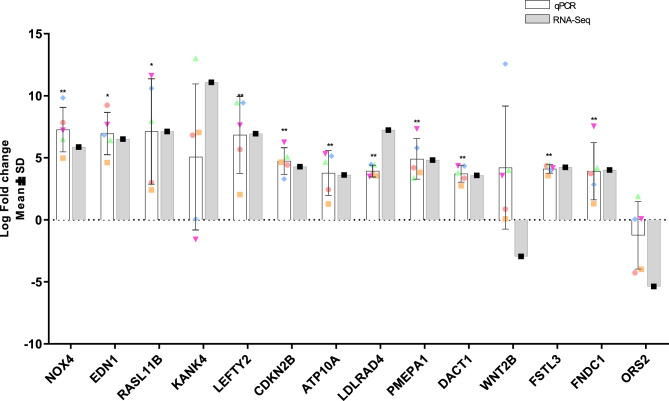
RT-qPCR confirmation and comparison of RNA-Seq results. A total of 5 control and TGFβ2 stimulated TM donor cells were used for the validation of gene expression patterns from the RNASeq analysis using RT-qPCR. Values were normalised to GAPDH and graphed as log fold change. Statistical significance was determined using a two-sample t test (*p < 0.0005).

To validate the results obtained from the RNA-Seq analysis, candidate genes with expression changes induce by TGF-β2 and pre-determined to regulate processes involved in glaucoma development and pathogenesis, were analysed from the same donors using RT-qPCR (Fig. 4). Thirteen of the fifteen candidate genes demonstrated significant differential expression by RT-qPCR with EDN1 (p < 0.04), RASL11B (p < 0.04), NOX4 (p < 0.003), LEFTY2 (p < 0.003), CDKN2B (P < 0.03), ATP10A (p < 0.002), LDLRAD4 (p < 0.005), FNDC1 (p < 0.002), PMEPA1 (p < 0.02) and DACT1 (p < 0.002) showing statistical significance. With the exception of WNT2B, the differential expression of the genes validated by RT-qPCR correlate to the expression patterns shown in the RNA-Seq data set, therefore confirming the reliability of the RNA-seq data.

## Discussion

Understanding the TGF-β2 induced genome-wide transcriptome changes in the TM cells has provided insights into the pathophysiological mechanisms driving primary open angle glaucoma (POAG). To our knowledge, this is the first report to use RNA-seq analysis to investigate the human TM transcriptome profile following TGF-β2 treatment. Understanding the transcriptome is essential for interpreting the functional elements of the genome and revealing the molecular constituents of cells and tissues, and for understanding development and disease^8,33,34^. Previous studies have performed gene expression studies using microarrays on cells derived from human glaucomatous donor eyes^28,35^. These studies are challenging as glaucomatous donor eyes and surgical samples are limited, the individual donors can have received different medical treatments prior to sampling, small amounts of RNA are available from surgical samples and POAG development is multifactorial and variable between patients^8,28,35,36^. Given the body of evidence implicating TGF-β2 in the development of POAG^9,12,19^, we employed a TGFβ2-induced TM cell culture model to mimic the elevated TGF-β2 environment of POAG, therefore identifying differentially expressed genes and associated pathways that contribute to the pathogenesis of POAG in a hypothesis-independent and discovery-driven manner. Understanding the role of TGF-β2 in the outflow pathway is essential to develop new therapies based on the modulation of TGF-β2 signalling in the trabecular meshwork^9,19,22^.

GWAS in glaucoma have identified multiple novel disease-predisposing genes^37^, but progress in uncovering the mechanisms by which these genes lead to glaucoma, a necessity to understanding disease pathogenesis and develop new therapies, has been considerably slower^38,39^. The differentially expressed genes (DEGs) and altered pathways identified in this study can provide further insight into the pathophysiological impact of genetic variants associated with POAG^3,8^. ATPase Phospholipid Transporting 10a (ATP10A) lies in a POAG mapped locus (GLC1I) on chr15q11-13^40^ and is upregulated by steroid treatment in the TM^41^. The association of genetic variants in CDKN2B with POAG has been replicated in multiple genome wide association studies^7,8,36^. CDKN2B encodes a cyclin-dependent kinase inhibitor, p15INK4b, which plays an important role in the regulation of the cell cycle through the inhibition of cyclin-dependent kinase 4 (CDK4)^42^. The expression of CDKN2B is significantly induced by TGF-β and plays a role in the mediation of TGF-β-induced cell cycle arrest^42–44^. TGF-β inhibits cell proliferation by producing G1 phase cell cycle arrest and CDKN2B, which forms a complex with either CDK4 or CDK6 preventing their activation, acts as an effector of TGF-β cell cycle arrest^45^. CDKN2B was amongst the common TGF-β2 stimulated DEGs in the previous microarray studies^23,24,46^ and in this RNA-Seq dataset. The upregulation of CDKN2B and CDKN2B-AS1 in human TM cells is associated with senescence^44^ and interestingly there is a decline in the TM cell population with age and this decline is accelerated in POAG^47,48^. The underlying mechanisms of TM cell loss with age and POAG have not been fully resolved and the interplay between TGF-β and TM senescence needs further investigation^40,44,49,50^^.^

Unsurprisingly, several of the DEGs induced by TGF-β2 in the TM have been implicated in the regulation of TGF-β2 signalling. Follistatin-like 3 (FSTL3) is a member of the follistatin family which includes follistatin (FST) and both can inhibit the actions of activins and bone morphogenic proteins (BMPs)^51^. BMPs are members of the TGF-β superfamily which have been identified in the human TM and BMPs can block TGF-β2 induction of ECM proteins in the TM^12,52^. The role of FSTL3 in glaucoma or following TGF-β2 induction in the TM is unknown, although FSTL3 has pro-fibrotic effects in cardiac fibroblasts, in partnership with connective tissue growth factor (CTGF)^53^. PMEPAI, LDLRAD4, SMAD7 and LEFTY2 are all negative regulators of TGF-β2 signalling^53–56^. Prostate transmembrane protein androgen induced 1 (PMEPA1) is a negative regulator of TGFβ signalling in prostate cancer cells^54^. Silencing SMAD7 in TM cells can attenuate the expression of ECM components induced by TGF-β2^57^. Left–right determination factor 2 (LEFTY2) was significantly upregulated in the TGF-β2 stimulated TM cells and negatively modulates both TGFβ and BMP signalling through the inhibition of R-Smad protein phosphorylation^56^. CTGF expression is induced by TGFβ, and while FSTL3 interacts with CTGF to drive fibrosis, LEFTY2 plays a significant role in the regulation of the ECM and the inhibition of the pro-fibrotic effects of CTGF^56,58^. CTGF is an important regulator and enhancer of TGF-β signalling in fibrosis which has been implicated in the pathogenesis of POAG^59,60^.

TM contractility is induced by endothelin 1 (EDN1), a potent vasoactive peptide, which has been linked to glaucoma pathogenesis in both humans and animal models^61,62^. Elevated expression of EDN1 in response to TGF-β2 was identified in this study and elevated EDN1 gene and protein expression has previously been shown in cultured trabecular meshwork cells treated with both TGF-β1 and -β2^63^. EDN1 has variable effects on IOP in animal models^61,63^ but *in-vitro* studies in bovine and human TM cells show that there is increased TM cell contractility in response to elevated EDN1 expression EDN1^64–67^; and this contractility can be reversed with Rho kinase inhibitors^66^. In podocytes TGF-β induces the synthesis and release of EDN1 which initiates mitochondrial ROS, mitochondrial DNA damage and mitochondrial dysfunction^68^. Similarly, there is an interplay between ROS and TGF-β in glaucoma^69^, and NOX4 is one of the major sources of cellular oxidative stress and is an important downstream effector in mediating TGF-β-induced fibrosis in the heart, lungs and kidneys via the production of ROS^70^. NOX4 was significantly upregulated by TGF-β2 in our study and has been demonstrated to drive ECM production, actin stress fibre formation and αSMA expression in the TM^71^.

Alongside the TGF-β signalling, the RhoA signalling pathway was also highlighted in both the GO and IPA pathway analysis as it had a many differentially expressed genes common to our data set and the regulatory pathway. The Rho pathway has pleiotropic functions including the regulation of cellular contraction, motility, morphology, polarity, cell division, apoptosis and gene expression^72^. In the anterior eye the Rho signalling pathway in combination with its major downstream effector, Rho-associated protein kinase (ROCK), modulate the cytoskeletal integrity of cells, synthesis of ECM components, and the permeability of cells in both the Schlemm’s canal and TM^72,73^. Several studies have highlighted the potential of ROCK inhibitors to reverse the physiological effects induced by TGF-β2, including reducing cell stiffness and HTM contractability^74,75^. Important therapeutic advances have thus been made with the ROCK inhibitor Ripasudil® approved for clinical use in glaucoma patients in Japan from 2014^76,77^. Following that, Rhopressa® was approved by the FDA in the USA in December 2017 for lowering the IOP in POAG patients^78^. Therapeutic agents targeting other TGF-β2 activated pathways in the TM highlighted in this RNA-seq dataset are worthy of further investigation. The nuclear factor (erythroid-derived 2)-like 2 (Nrf2), PI3K-Akt, MAPK and HIPPO signalling pathways have been implicated in POAG and TM patho-physiology, and were enriched in the pathway analysis supporting further study and therapeutic manipulation in glaucoma^79–82^.

In conclusion, this study presents a comprehensive characterisation of differentially expressed genes in response to TGF-β2 in the human trabecular meshwork and a mechanistic insight into the underlying biology of this disease. The trabecular meshwork plays a significant role in the regulation of outflow facility and intra-ocular pressure. Raised intra-ocular pressure is a major risk factor for primary open angle glaucoma, and the identification and characterisation of TGF-β2 dependent differentially expressed genes and pathways in human HTM cells is essential to understand the patho-physiology of glaucoma and to develop new therapeutic agents.

## Supplementary Information

Supplementary Table 1.

Supplementary Table 2.

Supplementary Figure 1.

Supplementary Figure 2.
