# Supplementary material for: Genome-wide transcriptome profiling of human trabecular meshwork cells treated with TGF-β2
Source: Sci Rep. 2022 Jun 10;12:9564. doi: 10.1038/s41598-022-13573-8 (PMC9187693; doi:10.1038/s41598-022-13573-8)
Supplement: Supplementary file 1 — Supplementary Table 1. [file 41598_2022_13573_MOESM1_ESM.pdf]

Supplemental Table S1: Summary of the Mapping Results for Each Sample.					
**Sample Name**	**Total Readcount**	**rRNA(%)**	**mtRNA(%)**	**Mapped(%)**	**Unmapped(%)**
CK_Group1	57023686	0.71	5.51	80.13	13.65
CK_Group2	44504091	0.52	2.21	80.21	17.05
CK_Group3	40024297	0.4	2.04	83.82	13.74
CK_Group4	47803322	0.27	4.12	82.29	13.32
CK_Group5	46181400	0.23	4.67	82.07	13.01
TK_Group1	47487795	0.57	4.51	80.4	14.52
TK_Group2	40939102	0.16	1.68	84.8	13.36
TK_Group3	55439358	0.38	1.62	84.05	13.96
TK_Group4	48512081	0.18	2.27	83.66	13.89
TK_Group5	44826263	0.81	3.94	82.06	13.19

Reads from each of the two libraries (Control_Group and Treatment_Group) were extracted based on their unique I.Ds. In total 40-57 million reads per sample were obtained. Based on alignment to the reference genome, *H.sapiens*, hg19 / GRC37, UCSC Genome Browser, the number of identified genes per sample was calculated. Reliability of identified genes increased with the number of identified fragments. All genes were included in statistical comparison of two groups, irrespective of how few calls have been made.
