# Supplementary material for: Genome-wide transcriptome profiling of human trabecular meshwork cells treated with TGF-β2
Source: Sci Rep. 2022 Jun 10;12:9564. doi: 10.1038/s41598-022-13573-8 (PMC9187693; doi:10.1038/s41598-022-13573-8)
Supplement: Supplementary file 2 — Supplementary Table 2. [file 41598_2022_13573_MOESM2_ESM.pdf]

Supplemental Table S2: Primer sequences for qPCR	
**Primer Name**	**Sequence**
EDN1	**F** 5′-CCAGGAGCTCCAGAAACAG-3′<br>**R** 5′-GAGCAGGAGCAGCGCTT-3′
NOX4	**F** 5′-CACAGACTTGGCTTTGGATTTC-3′<br>**R** 5′-GGATGACTTATGACCGAAATGATG-3′
FSTL3	**F** 5′-GACTTCATCAGGAACAAGTGGTC-3′<br>**R** 5′-AGGTCCGTAGCCATGAGGAT-3′
FNDC1	**F** 5′-GACTTCATCAGGAACAAGTGGTC-3′<br>**R** 5′-AGGTCCGTAGCCATGAGGAT-3′
KANK4	**F** 5′-CATCTTCAGCCTTGAATTCCTCAT-3′<br>**R** 5′-TTCTAATGTGCTAATGTGCTCCTG-3′
ATP10A	**F** 5′-TCGGCATTGTCATCTACGCA-3′<br>**R** 5′-AGGAGCAGGACACACCAGA-3′
WNT2B	**F** 5′-TTGGAGTGGTAGCCATAAGCAT-3′<br>**R** 5′-TTGAACGCTGACTGTGTAGGT-3′
LDLRAD4	**F** 5′-AAGAGTTGGAGCACAGGCTT-3′<br>**R** 5′-TTACAGACCAGCGAACCAAGA-3′
PMEPA1	**F** 5′-TTAGACTCCGCTCTTGTTCTCC-3′<br>**R** 5′-ATGCTCTCCTCTGGTCACCT-3′
CDKN2B	**F** 5′-GCAAGCCTGTCTGAGACTCA-3′<br>**R** 5′-ACACACTCCTAAATATCCCTGGAA-3′
CDKN2B-AS1	**F** 5′-CCACATCAATGATGAAGCCAGAA-3′<br>**R** 5′-TTGATCTCTGCTGTTGAATCAGAAT-3′
RASL11B	**F** 5′-CCTCACCAAACGATTCATCGG-3′<br>**R** 5′-ACCTGGAGTGTCTTGAACCTG-3′
OSR2	**F** 5′-TCTCCACACAAATGTCCCACA-3′<br>**R** 5′-TTTCGCCTGAACACTTTGCC-3′
LEFTY2	**F** 5′-GGTGGTCTTAATGTAGGTCTTAACTT-3′<br>**R** 5′-AGACAGGAAATGGAAGGACACA-3′
DACT1	**F** 5′-CTTCATGCTGTGGCTGTGC-3′<br>**R** 5′-GGACGGTAAGGAACTGTCTGT-3′
GAPDH	**F** 5′-CGAGCCACATCGCTCAGACACC-3′<br>**R** 5′-GGTCAATGAAGGGGTCATTGATGGCAAC-3′
