# Supplementary material for: Genome-wide transcriptome profiling of human trabecular meshwork cells treated with TGF-β2
Source: Sci Rep. 2022 Jun 10;12:9564. doi: 10.1038/s41598-022-13573-8 (PMC9187693; doi:10.1038/s41598-022-13573-8)
Supplement: Supplementary file 3 — Supplementary Figure 1. [file 41598_2022_13573_MOESM3_ESM.pdf]

A

Donor I.D	Age	Sex	Medical History
Donor A	57	M	No Glaucoma History
Donor B	65	M	No Glaucoma History
Donor C	57	M	No Glaucoma History
Donor D	57	M	No Glaucoma History
Donor E	64	M	No Glaucoma History

B

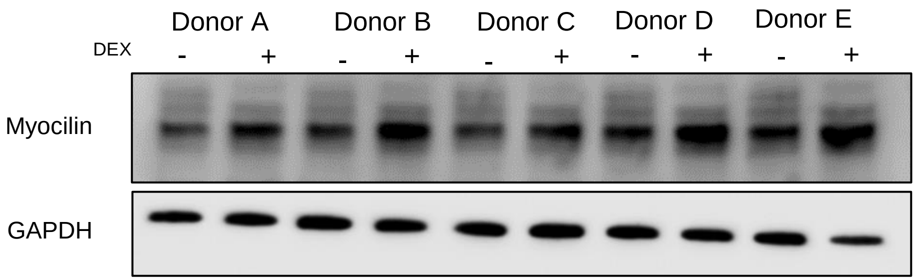

C

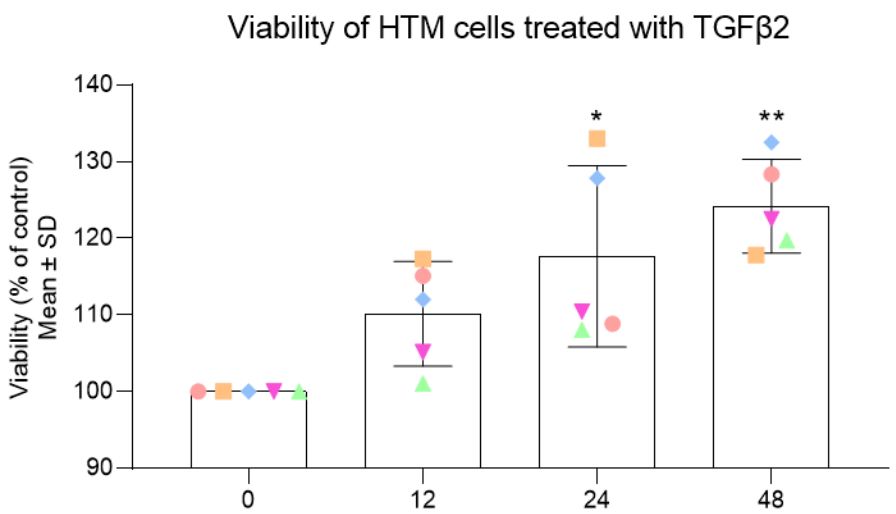

Supplementary figure 1:(A) Age and gender characteristics of donors used in this study. Eyes were obtained from the Royal Liverpool University Hospital Mortuary and medical history was unknown. Donor eyes were excluded if the maximum post-mortem time exceeded 48 hours or there was a history of glaucoma or ocular surgery. (B) Characterisation of normal human TM cells from donors. Donor TM cells were treated with or without 100mM dexamethasone for 10 days and a western blot performed to detect myocilin upregulation. (C) Cell viability of TM cells treated with TGFβ2, assessed using a MTT assay, over 12, 24 and 48 hours. Statistical significance was determined using a one-way ANOVA with Dunnett's post-hoc multiple comparisons test.
