# Supplementary material for: Genome-wide transcriptome profiling of human trabecular meshwork cells treated with TGF-β2
Source: Sci Rep. 2022 Jun 10;12:9564. doi: 10.1038/s41598-022-13573-8 (PMC9187693; doi:10.1038/s41598-022-13573-8)
Supplement: Supplementary file 4 — Supplementary Figure 2. [file 41598_2022_13573_MOESM4_ESM.pdf]

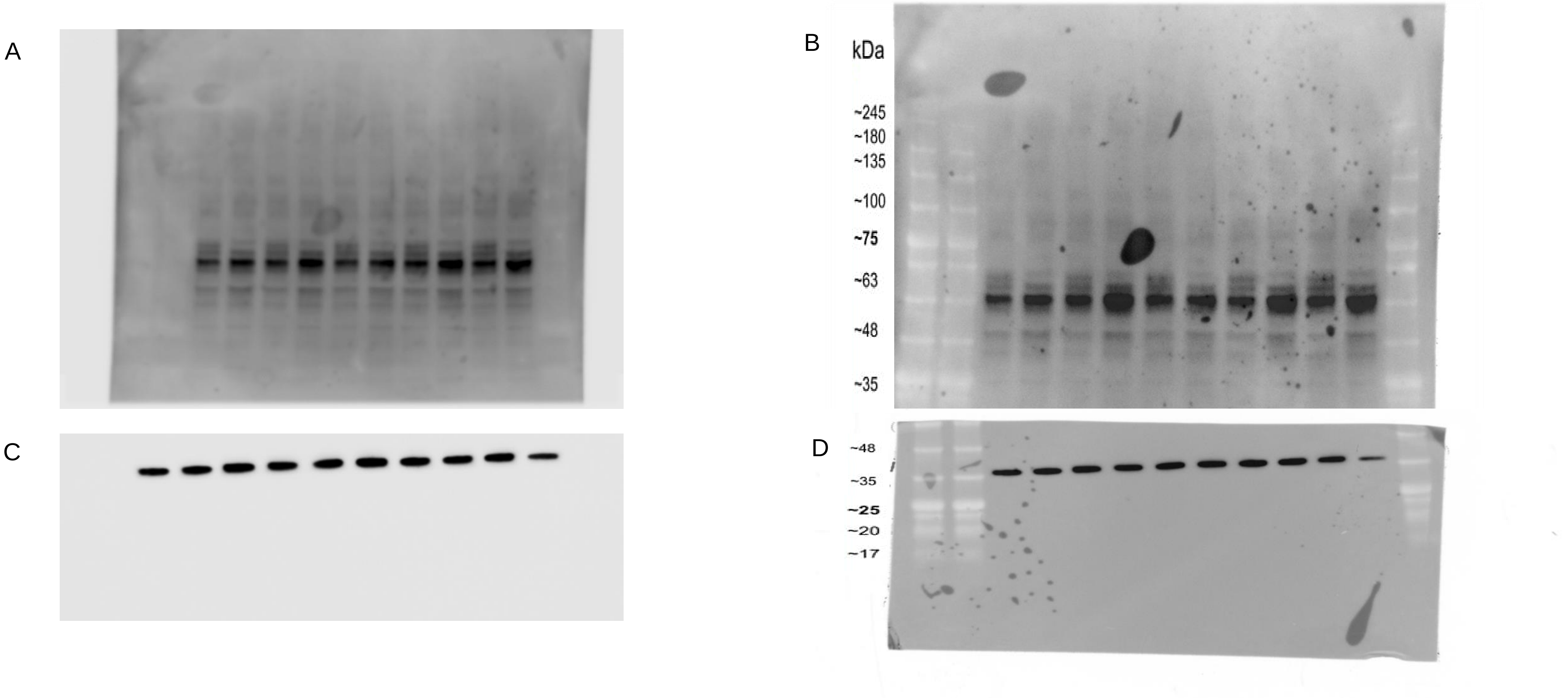

Supplementary figure 2: Original blots: A – Myocilin blot, B – Myocilin blot with visible marker, C - GAPDH loading control blot, D - Myocilin blot with visible marker.
